# Supplementary figures and images for: Mycobacterium tuberculosis Infection among 1,659 Silicosis Patients in Zhejiang Province, China
Source: Microbiol Spectr. 2022 Dec 1;10(6):e01451-22. doi: 10.1128/spectrum.01451-22 (PMC9784764; doi:10.1128/spectrum.01451-22)

**Supplemental Figure 1. MTB status in patients with different silicosis categories**

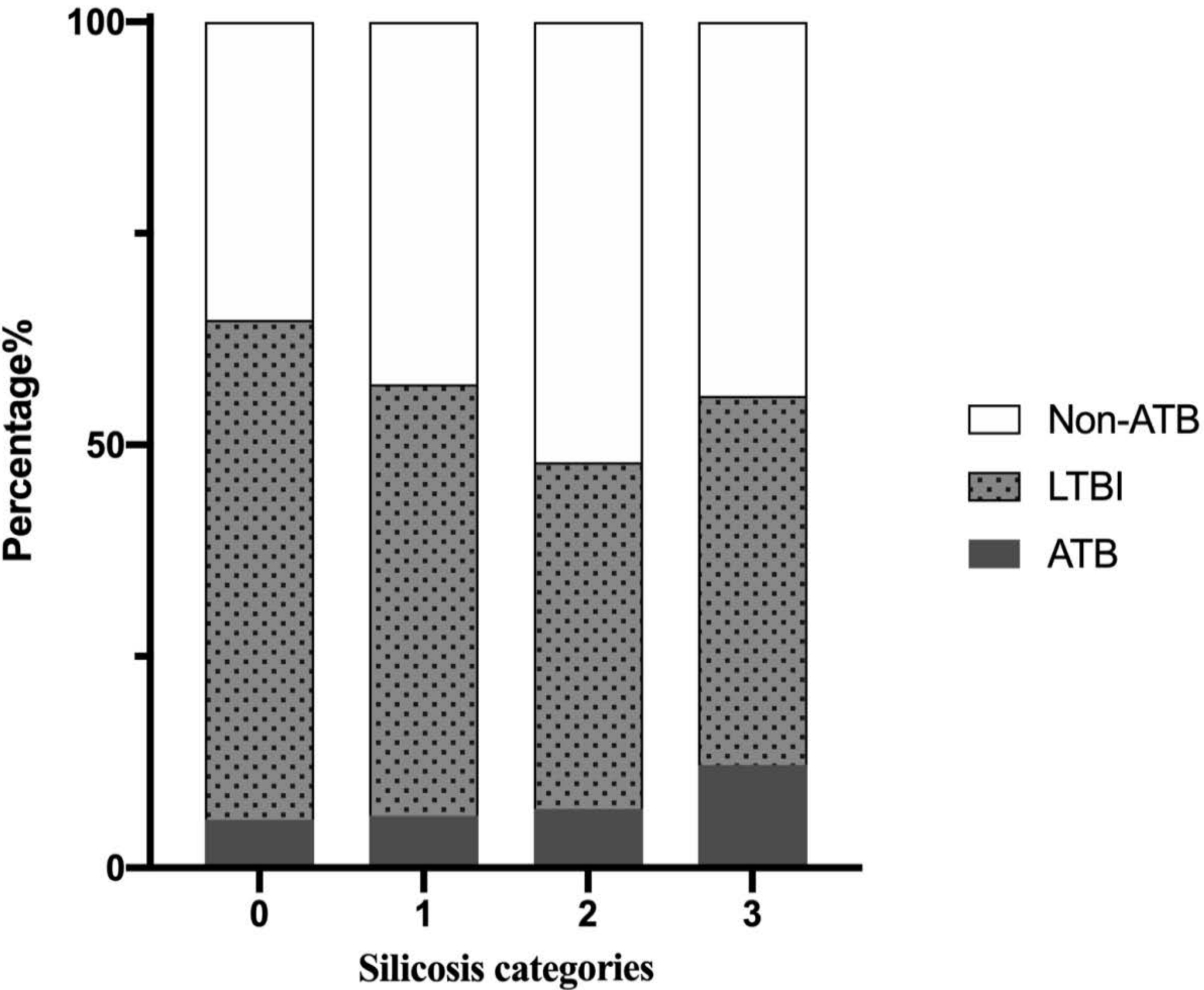

Supplement: Supplemental file 1 — Supplemental material. Download spectrum.01451-22-s0001.pdf, PDF file, 0.1 MB [file spectrum.01451-22-s0001.pdf]
